# Resistance Exercise Training Selectively Modulates Tumor Suppressor and Muscle-Regulatory MicroRNAs in Breast Cancer Survivors and Healthy Postmenopausal Women: An Exploratory Study

**DOI:** 10.3390/ijms27177900

**Published:** 2026-09-04

**Authors:** Macarena Artigas-Arias, Josefa Bonicioli, Nolberto Huard, Luis A. Salazar, Jorge Sapunar, Luis Peñailillo, Denisse Valladares-Ide, Rui Curi, Gabriel Nasri Marzuca-Nassr

**Affiliations:** 1 Doctorado en Ciencias Mención Biología Celular y Molecular Aplicada, Universidad de La Frontera, Temuco 4811230, Chile; macarena.artigas@falp.org; 2 Centro de Investigación e Innovación del Cáncer, Fundación Arturo López Pérez OECI Cancer Center, Santiago 7500921, Chile; jorge.sapunar@falp.org; 3 Exercise and Rehabilitation Sciences Institute, School of Physical Therapy, Faculty of Rehabilitation Sciences, Universidad Andres Bello, Santiago 7591538, Chile; j.bonicioliprez@uandresbello.edu (J.B.); luis.penailillo@unab.cl (L.P.); 4 Center of Molecular Biology and Pharmacogenetics, Department of Basic Sciences, Faculty of Medicine, Universidad de La Frontera, Temuco 4811239, Chile; nolberto.huard@ufrontera.cl (N.H.); luis.salazar@ufrontera.cl (L.A.S.); 5 Departamento de Medicina Interna, Facultad de Medicina, Universidad de La Frontera, Temuco 4811239, Chile; 6 Instituto de Ciencias de la Salud, Universidad de O’Higgins, Rancagua 2820000, Chile; denisse.valladares@uoh.cl; 7 Interdisciplinary Post-Graduate Program in Health Sciences, Institute of Physical Activity and Sports, Universidade Cruzeiro do Sul, São Paulo 01506-000, Brazil; ruicuri59@gmail.com; 8 Educantion Center, Butantan Institute, São Paulo 05508-040, Brazil; 9 Postgraduate Program in Structural and Functional Interactions in Rehabilitation, University of Marília (UNIMAR), Marília 17525-900, Brazil; 10 Departamento de Ciencias de la Rehabilitación, Facultad de Medicina, Universidad de La Frontera, Temuco 4811239, Chile

**Keywords:** breast cancer, hormone therapy, strength training, microRNAs

## Abstract

Progressive resistance exercise training (RET) enhances skeletal muscle mass in healthy postmenopausal women (HEAs) and breast cancer survivors (BCSs) undergoing endocrine therapy. MicroRNAs (miRNAs) are recognized as key epigenetic regulators of exercise-induced adaptations, functioning as tumor suppressors, oncomiRs, and modulators of skeletal muscle homeostasis. Nevertheless, the impact of RET on circulating miRNA profiles in this population remains largely unexplored. This study aimed to explore the effects of a 12-week progressive RET program on plasma miRNAs associated with tumor biology and skeletal muscle regulation in HEAs and BCSs. Five HEAs and five BCSs undergoing endocrine therapy completed a 12-week supervised progressive RET program (3 sessions/week, 60–80% 1RM). Plasma samples were collected before and after intervention, and candidate miRNA expression was quantified by qRT-PCR, normalized to hsa-miR-16-5p, and analyzed using the 2^−ΔΔCt^ method. At baseline, no significant differences were observed between HEA and BCS in tumor suppressor miRNAs (miR-let-7f-5p, miR-125a-5p, miR-342-3p), oncomiRs (miR-21-5p, miR-155-5p, miR-221-3p), or skeletal muscle–related miRNAs (miR-206-3p, miR-486-5p) (*p* > 0.05). The only exception was miR-375-3p, which showed increased expression in HEA vs BCS (*p* = 0.037). After 12 weeks of RET, The BCS group showed increases in miR-125a-5p (*p* = 0.050) and miR-342-3p (*p* = 0.048) with significant group × time interactions that remained significant after Benjamini–Hochberg False Discovery Rate (BH-FDR) correction (FDR-adjusted *p* = 0.042 for both). Both groups displayed a nominal increase in miR-375-3p (*p* = 0.042), which did not remain significant after FDR correction. No significant changes were detected for miR-let-7f-5p or the analyzed oncomiRs in either group. Both miR-206-3p and miR-486-5p exhibited upward trends in the BCS group (*p* > 0.05). In this exploratory study, 12-week RET program was associated with selective changes in plasma miRNA expression in the HEA and BCS groups, suggesting that this training regimen may act as an epigenetic modulator of circulating miRNAs involved in tumor suppression and skeletal muscle homeostasis.

## 1. Introduction

Estrogen receptor–positive (ER+) breast cancer represents the most prevalent subtype, accounting for approximately 70% of cases and primarily affecting postmenopausal women [1]. Advances in diagnosis and treatment, particularly adjuvant endocrine therapy based on the inhibition of estrogen production or action through selective estrogen receptor modulators (SERMs), such as tamoxifen, and/or aromatase inhibitors (AIs), including anastrozole, letrozole, and exemestane, have significantly improved survival and reduced the risk of disease recurrence [2,3,4,5]

Nevertheless, these clinical benefits shown by SERMs are accompanied by significant adverse effects on musculoskeletal health and body composition, including skeletal muscle mass loss, reduced muscle strength, decreased bone mineral density, and increased central adiposity; effects that are further exacerbated by the postmenopausal state [1,6,7,8,9]. Sustained estrogen suppression and cancer-related metabolic alterations promote a catabolic environment, characterized by mitochondrial dysfunction in skeletal muscle and increased fatigue, ultimately contributing to reduced exercise tolerance and functional decline [8,9]. At the molecular level, endocrine therapy induces skeletal muscle mass loss by suppressing anabolic estrogen signaling and activating FOxO -and NF-kB- dependent catabolic pathways [10]. This process is further exacerbated by increased oxidative stress mediated by TGF-β/Nox4, impaired excitation–contraction coupling, and reduced muscle-regenerative capacity, culminating in atrogene activation and progressive muscle atrophy [9].

Within this framework, skeletal muscle mass decline emerges as a clinically meaningful factor, as it has been associated with worse oncological and functional outcomes, including increased risk of tumor recurrence [11], reduced tolerance to endocrine therapy [12], and higher mortality among breast cancer survivors [13]. For instance, Zhang et al. (2020) reported in a systematic review and meta-analysis that women with early-stage breast cancer who exhibited lower-than-expected skeletal muscle mass for their age had a significantly higher risk of all-cause mortality compared with those with preserved muscle mass, independent of age and tumor stage [13]. Furthermore, recent clinical data extend these findings to treatment adherence, as Saraf et al. (2024) reported that sarcopenic women with early-stage hormone receptor–positive breast cancer experienced higher rates of toxicity-related treatment modifications and premature discontinuation of adjuvant endocrine therapy, resulting in lower rates of treatment completion [10]. In parallel, endocrine therapy resistance affects approximately 30–50% of patients, limiting long-term treatment efficacy and contributing to disease recurrence [11,14]. Together, these findings suggest that intrinsic biological resistance and reduced treatment exposure associated with skeletal muscle deterioration may coexist and collectively contribute to poor oncological outcomes. Thus, preserving skeletal muscle mass during endocrine therapy has emerged as a clinically relevant strategy to improve treatment tolerance and long-term outcomes in breast cancer survivors. However, the molecular mechanisms by which exercise preserves skeletal muscle and tumor homeostasis during endocrine therapy remain unclear.

Physical exercise is an effective non-pharmacological intervention for this population [15], with benefits in cardiorespiratory fitness [16], muscle strength [17], and psychological well-being [18], as well as reductions in cancer-specific mortality, overall mortality, and recurrence [19]. These effects appear to be mediated by improved insulin sensitivity, and hormonal regulation, reduced systemic inflammation, and enhanced immune surveillance, including the transient mobilization of T lymphocytes and natural killer (NK) cells [20]. Among exercise modalities, progressive resistance exercise training (RET) is particularly relevant for preserving skeletal muscle mass and counteracting menopause -and oncologic therapy-induced muscle atrophy [21].

Beyond clinical effects, recent research has highlighted the role of epigenetic mechanisms in mediating exercise benefits. Among these, microRNAs (miRNAs), small non-coding RNAs (~20 nucleotides), have emerged as key regulators that modulate post-transcriptional gene expression and participate in essential cellular processes such as proliferation, apoptosis, and angiogenesis [22]. In skeletal muscle, muscle-specific or muscle-enriched miRNAs known as (myomiRs), including miR-1, miR-133a, miR-206, and miR-486, can be detected in plasma and have been identified as key regulators of muscle proliferation, differentiation, and regeneration [23]. These plasma miRNAs modulate central pathways involved in muscle homeostasis, such as the IGF-1/Akt/mTOR pathway, which promotes protein synthesis and the FOxO/ubiquitin–proteasome system/autophagy pathway, which is associated with protein degradation. For instance, reduced expression of miR-1 and miR-133a favors IGF-1 activation and enhances protein synthesis, whereas downregulation of miR-206 increases IGF-1 expression and further potentiates anabolic signaling [24]. Conversely, miR-486 negatively regulates PTEN and FOxO1, promoting Akt phosphorylation and anabolic activation, while miR-486 inhibition activates genes involved in muscle atrophy [25]. Collectively, these findings underscore that miRNAs function as critical epigenetic checkpoints that dynamically regulate muscle protein synthesis and degradation, thereby maintaining skeletal muscle homeostasis in breast cancer survivors [26].

Interestingly, the regulatory role of miRNAs in homeostasis is not limited to maintaining skeletal muscle mass to also encompasses tumor homeostasis [27]. Accordingly, beyond myomiRs, several circulating miRNAs traditionally associated with cancer biology also regulate both muscular and tumoral processes [28]. Some of these, such as let-7f-5p, miR-125a-5p, miR-342-5p, and miR-375-3p, function as tumor suppressor miRNAs by inhibiting cell proliferation and negatively regulating oncogenic signaling pathways [29]. In contrast, oncomiRs, including miR-155-5p, miR-21-5p, and miR-221-3p, promote tumor progression and apoptosis resistance [30]. Thus, because of their dual regulatory role and remarkable stability in biological fluids, miRNAs arepromising non-invasive biomarkers with diagnostic, prognostic, and therapeutic relevance [31].

Preclinical evidence indicates that exercise can modulate circulating miRNAs involved in both tumor biology and skeletal muscle regulation. In breast cancer animal models, increases in miR-206 and let-7, identified as tumor suppressor miRNAs, along with reductions in the cancer-associated miRNA (oncomiR) miR-21 were reported following five weeks of high-intensity interval training (HIIT) [32]. In humans, although studies are limited, interventions combining weight loss with moderate-intensity aerobic exercise over 6 months [33], as well as 12-week HIIT programs [34], have demonstrated changes in circulating miRNA profiles related to tumor suppression in breast cancer survivors receiving endocrine therapy. However, the specific effects of progressive RET on miRNAs involved in muscle and tumor regulation in this population remain poorly understood [35]. Therefore, the present exploratory study aimed to evaluate changes in the plasma expression of selected miRNAs associated with tumor and skeletal muscle regulation in postmenopausal breast cancer survivors undergoing endocrine therapy and in healthy postmenopausal women, following a 12-week progressive RET program. This approach seeks to provide initial mechanistic evidence on the role of miRNAs as molecular mediators of exercise-induced adaptations and their potential relevance for recurrence prevention and the preservation of skeletal muscle mass in this vulnerable population.

## 2. Results

### 2.1. Participant Characteristics

Table 1 shows the baseline characteristics of the study participants. Five healthy postmenopausal women (HEA) and five postmenopausal non-metastatic breast cancer survivors (BCS) with hormone receptor–positive disease undergoing adjuvant endocrine therapy were included. No statistically significant differences were observed between groups in age, anthropometric and functional characteristics, physical activity level, or nutritional status.

These microRNAs were previously identified through a targeted literature review as key regulators of tumor biology and skeletal muscle-related pathways.

### 2.2. Tumor Suppressor microRNA Expression

**Figure 1:**
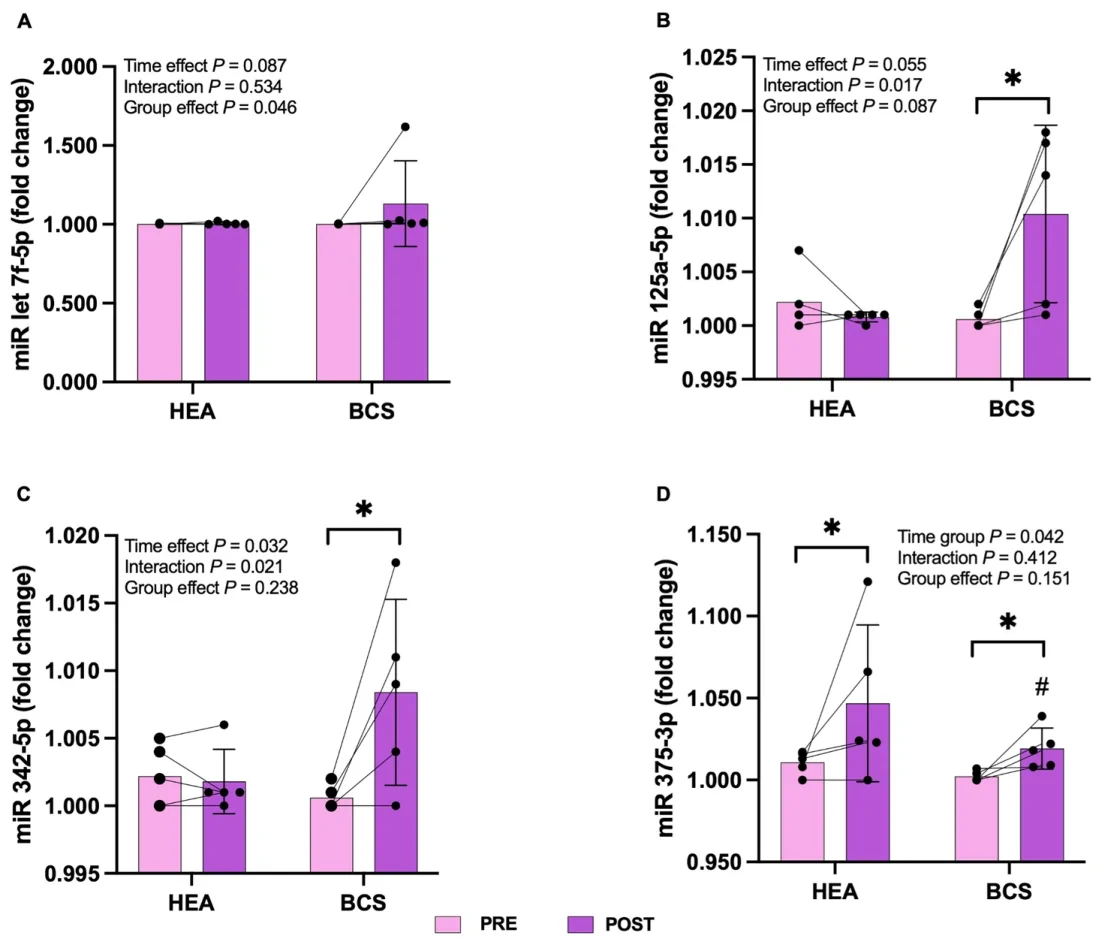
Expression of tumor suppressor microRNAs. Plasma expression levels of (**A**) miR-let-7f-5p, (**B**) miR-125a-5p, (**C**) miR-342-5p, and (**D**) miR-375-3p before (PRE) and after (POST) 12 weeks of resistance exercise training in the HEA (n = 5) and BCS (n = 5) groups. Data were analyzed using two-way repeated-measures ANOVA (time × group). The symbol * indicates a main effect of time (*p* < 0.05), corresponding to significant changes between PRE and POST irrespective of group; # indicates a significant difference between groups at baseline. Fold change represents relative expression. Values are normalized to the reference miRNA hsa-miR-16-5p.

The results indicate that no statistically significant differences were observed in baseline expression levels of tumor suppressor miRNAs (miR-let-7f-5p, miR-125a-5p, and miR-342-3p) between the HEA and BCS groups (*p* > 0.05). The only exception was miR-375-3p, which exhibited significantly higher baseline expression in the HEA group than in the BCS group (*p* = 0.037) (Figure 1).

Regarding the relative expression of miR-let-7f-5p, no significant changes were observed after 12 weeks of RET (time effect, *p* = 0.308). Expression levels remained stable in both groups (HEA: 1.0022 ± 0.0038 to 1.0054 ± 0.0008; BCS: 1.0030 ± 0.0001 to 1.1320 ± 0.2719), with no evidence of a group × time interaction (*p* = 0.118; Figure 1A).

In contrast, after 12 weeks of RET, we observed differential regulation of specific miRNAs between groups was observed. Two-way repeated-measures ANOVA revealed a significant group × time interaction for miR-125a-5p expression (Figure 1B; *p* = 0.017; η^2^ = 0.53), indicating a differential response between groups over time. Post hoc analyses showed that miR-125a-5p expression increased after 12 weeks of RET in the BCS group (*p* = 0.050), whereas no significant changes were observed in the HEA group (*p* = 0.325). When comparing the magnitude of relative change (%Δ) between groups, an independent Student’s *t*-test revealed a significant difference (Supplementary Figure S1B; *p* = 0.032), with an average increase of +1.02 ± 0.8% in the BCS group and no significant variation in the HEA group (−0.14 ± 0.3%).

A similar pattern was observed for miR-342-3pexpression, which also showed a significant group × time interaction (Figure 1C; *p* = 0.021; η^2^ = 0.49), indicating a differential response to training between the BCS and HEA groups. Specifically, post hoc analyses demonstrated that miR-342-3p expression increased after 12 weeks of RET in the BCS group (*p* = 0.048), whereas no relevant changes were observed in the HEA group (*p* = 0.621). Between-group comparisons of the relative change (%Δ) between groups showed a significant difference (Supplementary Figure S1C; *p* = 0.021), with an average increase of +0.86 ± 0.6% in BCS and no significant variation in HEA (−0.04 ± 0.2%). Importantly, the group × time interactions for miR-125a-5p and miR-342-3p remained significant after Benjamini-Hochberg False Discovery Rate (BH-FDR) correction (FDR-adjusted *p* = 0.042 for both).

Regarding miR-375-3p, relative expression increased significantly after 12 weeks of RET in both groups, with an average increase of +3.6 ± 2.1% (1.0108 ± 0.0070 to 1.0468 ± 0.0478) in the HEA group and +1.7 ± 0.6% (1.0022 ± 0.0032 to 1.0192 ± 0.0126) in the BCS group (nominal time effect *p* = 0.042; η^2^ = 0.42), with no between-group differences (group × time interaction *p* = 0.418; group effect *p* = 0.151; Figure 1D). However, this time effect did not remain statistically significant after BH-FDR correction (FDR-adjusted *p* = 0.073). Consistently, the percentage change did not differ significantly between the HEA and BCS groups (Supplementary Figure S1D; independent *t*-test *p* > 0.05). The nominal and FDR-adjusted *p*-values for the tumor-suppressor miRNA analyses are provided in Supplementary Table S1.

### 2.3. Expression of oncomiRs

**Figure 2:**
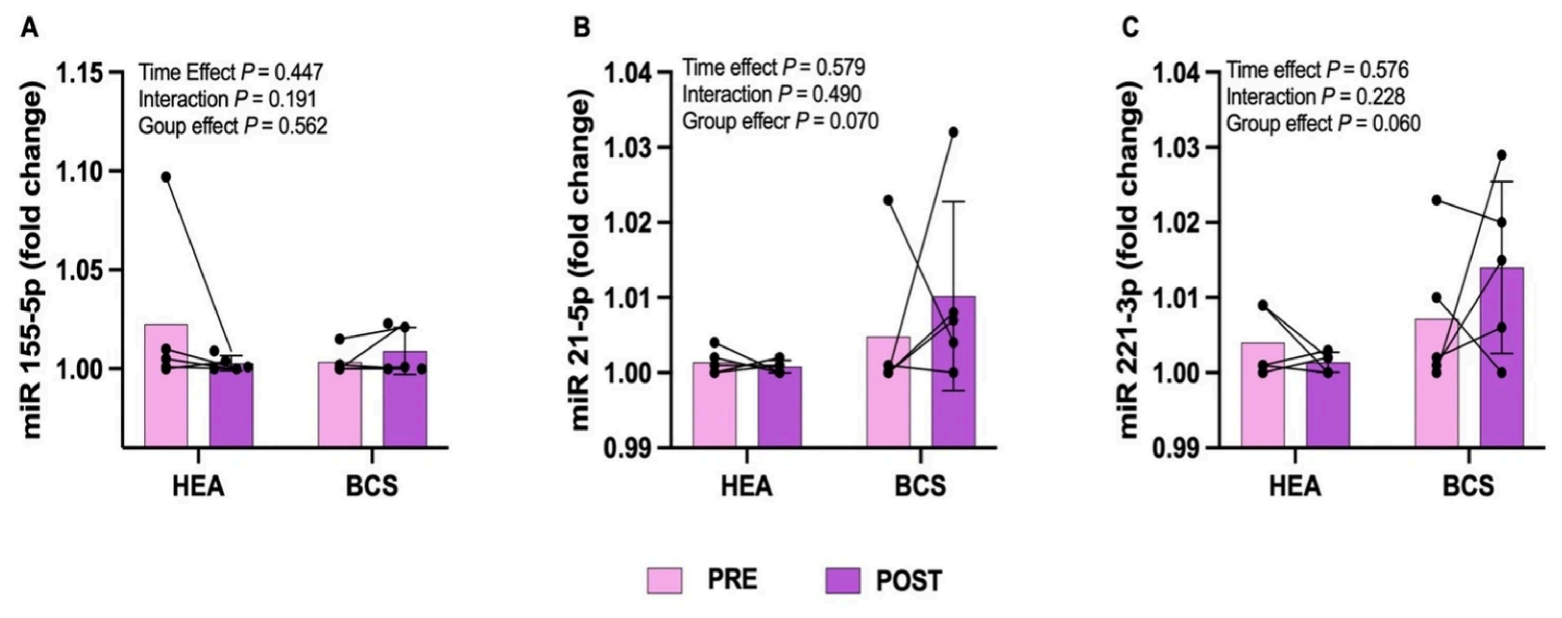
OncomiRNA expression. Plasma expression levels of (**A**) miR-155-5p, (**B**) miR-21-5p, and (**C**) miR-221-3p before and after 12 weeks of resistance exercise training in the HEA (n = 5) and BCS (n = 5) groups. Data were analyzed using repeated-measures ANOVA, with no significant interaction effects or between-group differences observed. Fold change: relative change. HEA: healthy women group; BCS: breast cancer survivor group. Values are expressed relative to hsa-miR-16-5p as the reference gene.

No differences in expression levels at baseline of the evaluated oncomiRs (miR-155-5p, miR-21-5p, and miR-221-3p) between the HEA and BCS groups (*p* > 0.05 for all comparisons; Figure 2).

After 12 weeks of RET, we observed no significant differences were observed in the expression of the evaluated oncomiRs between the groups (time effect, *p* > 0.05). Specifically, relative expression of miR-155-5p remained stable in both (HEA: 1.0226 ± 0.0418 to 1.0028 ± 0.0038; BCS: 1.0034 ± 0.0065 to 1.0090 ± 0.0119; time effect, *p* = 0.447), with no evidence of a group × time interaction (*p* = 0.191; Figure 2A). Analysis of the magnitude of relative change (%Δ) showed an average variation of −1.82 ± 1.60% in HEA and +0.52 ± 0.40% in BCS, indicating a slight trend toward decreased expression only in the HEA. However, between-group comparisons showed no statistically significant differences (*p* = 0.189).

Similarly, relative expression of miR-21-5p remained unchanged after 12 weeks of RET (HEA: 1.0014 ± 0.0001 to 1.0008 ± 0.0008; BCS: 1.0048 ± 0.0102 to 1.0102 ± 0.0126; time effect, *p* = 0.579), with no evidence of a group × time interaction (*p* = 0.490; Figure 2B). Between-group comparisons also showed no statistically significant differences (*p* = 0.481). Consistently, relative expressions of miR-221-3p did not show significant changes following the intervention (HEA: 1.0040 ± 0.0046 to 1.0014 ± 0.0001; BCS: 1.0072 ± 0.0097 to 1.0140 ± 0.0114; time effect, *p* = 0.576), with no evidence of a group × time interaction (*p* = 0.228; Figure 2C). Likewise, between-group comparisons shoed no statistically significant differences (*p* = 0.271).

### 2.4. Expression of miRNAs Involved in Skeletal Muscle Regulation

**Figure 3:**
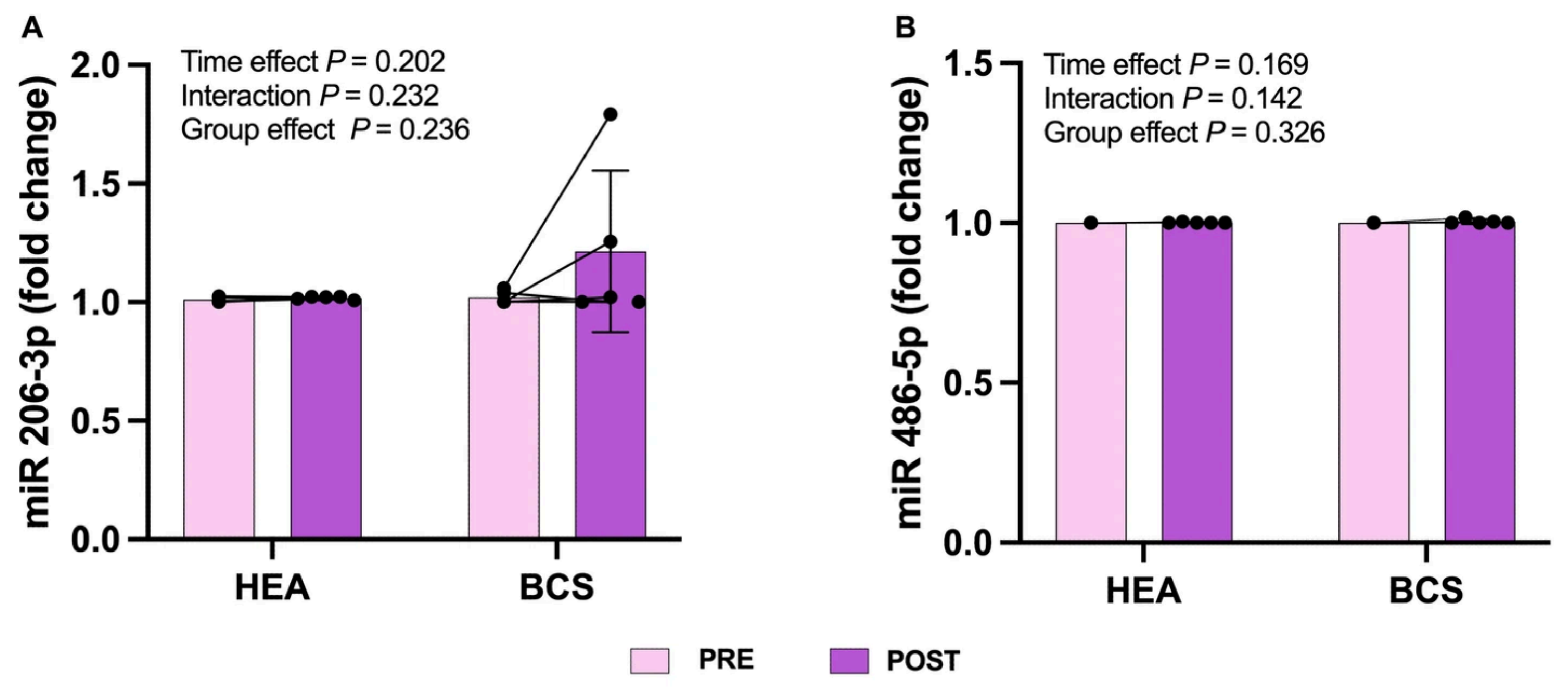
Expression of microRNAs involved in skeletal muscle regulation. Plasma expression levels of (**A**) miR-206-3p and (**B**) miR-486-5p before and after 12 weeks of resistance exercise training in the HEA (n = 5) and BCS (n = 5) groups. Data were analyzed using repeated-measures ANOVA (time × group), with no significant interaction or between-group differences observed. Fold change represents relative expression. HEA: healthy postmenopausal women; BCS: breast cancer survivors Values are expressed relative to hsa-miR-16-5p as the reference gene.

No differences were observed at baseline in the expression levels of miRNAs associated with skeletal muscle mass regulation (miR-206-3p and miR-486-5p) between HEA and BCS (*p* > 0.05; Figure 3).

After a 12-week RET program, no significant changes in miR-206-3p expression were observed in either group (HEA: 1.0102 ± 0.0120 to 1.0172 ± 0.0064; BCS: 1.0200 ± 0.0274 to 1.2140 ± 0.3413; time effect *p* = 0.202), with no evidence of a group × time interaction (*p* = 0.232; Figure 3A). When analyzing the magnitude of relative change (%Δ), the HEA group showed and an average increase of +1.72 ± 0.61%, and the BCS group showed +21.4 ± 15.3%, indicating a trend toward increased expression in both. However, between-group comparisons using an independent Student’s T-test showed no significant differences (*p* = 0.261). Similarly, relative expression of miR-486-5p remained unchanged following the intervention (HEA: 1.0004 ± 0.0055 to 1.0010 ± 0.0173; BCS: 1.0004 ± 0.0005 to 1.0048 ± 0.0075; time effect *p* = 0.169), with no evidence of a group × time interaction (*p* = 0.283; Figure 3B). Between-group comparisons using an independent Student’s T-test also showed no statistically significant differences (*p* = 0.283).

## 3. Discussion

This exploratory study investigated whether a 12-week progressive RET program was associated with changes in plasma expression of miRNAs involved in tumor and skeletal muscle regulation in healthy postmenopausal women and postmenopausal breast cancer survivors receiving endocrine therapy. Our findings demonstrate selective changes in the circulating expression of specific tumor-suppressive miRNAs following RET, with distinct response patterns between groups. Specifically, miR-125a-5p and miR-342-3p showed significant group × time interactions, which remained significant after BH-FDR correction, with post hoc analyses indicating increases exclusively in the BCS group. In addition, miR-375-3p showed a nominally significant increase over time in both groups, although this effect did not remain significant after FDR correction. In contrast, no significant changes were detected in the expression of the evaluated oncomiRs or myomiRNAs. These findings provide preliminary evidence that RET is associated with favorable molecular responses involving miRNAs linked to tumor-suppressive pathways.

We found that miR-125a-5p increased after RET (Figure 1B). Interestingly, a previous study reported that miR-125a-5p expression levels were lower in breast cancer patients with short survival (<1 year) compared to those with prolonged survival (>5 years) [36]. This finding suggests a relevant prognostic role for miR-125a-5p, whose downregulation may be associated with more aggressive tumor progression.

At the molecular level, miR-125a-5p directly inhibits the expression of HDAC4, a histone deacetylase that represses pro-apoptotic genes such as Bcl-2–associated X protein (BAX), BH3-interacting domain death agonist (BID), p53 upregulated modulator of apoptosis (PUMA), and p21. Thus, previous mechanistic evidence suggests that dysregulation of this pathway could favor cellular survival and tumor resistance [37]. In vitro models have shown that overexpression of miR-125a-5p reduces breast cancer cell proliferation and motility, and low serum levels have been correlated with poorer survival outcomes, supporting its potential as prognostic biomarker [36].

In the present study, the increase in miR-125a-5p following RET in BCS represents a favorable exercise-associated molecular response consistent with the previously described tumor-suppressive functions of this miRNA. In this sense, it may point to regulatory pathways relevant to tumor suppression [38]. Mareover, recent studies have shown that RET-activated skeletal muscle releases myokines with direct antitumor effects that inhibit proliferation and promote apoptosis in breast cancer cells in vitro [39]. For example, it wasreported that 12 weeks of RET combined with whole-body electromyostimulation in women undergoing antineoplastic treatment for breast cancer increased the secretion of myokines such as CXCL1, IL-10, and CCL4, thereby promoting caspase-3/7 activation and enhancing apoptosis in breast cancer cell lines. In that study, applying post-training serum to tumor cell cultures reduced cellular proliferation and increased programmed cell death, thus confirming a direct antitumor effect mediated by activated skeletal muscle [39]. These findings support the hypothesis that muscle contraction during RET may induce a systemic antitumor microenvironment by releasing myokines and modulating tumor-suppressive miRNAs, warranting futher mechanistic investigation.

Another miRNA with well-established tumor-suppressive properties is miR-342-3p, which has been reported to inhibit breast cancer cell proliferation, migration, and invasion. Importantly, we observed a significant increase in miR-342-3p expression following RET in the BCS group (Figure 1C), suggesting that resistance is associated with a molecular response consistent with this miRNA’s previously described tumorsuppressive functions.

Bioinformatic analyses have predicted miR-342-3p binding sites within the 3′UTR region of inhibin subunit beta A (INHBA) mRNA, a member of the TGF-β superfamily implicated in the progression of multiple neoplasms, including gastric cancer, nasopharyngeal carcinoma, and ovarian cancer [40,41,42]. In this regard, INHBA has been validated as a potential prognostic marker in breast cancer [43]. Its downregulation has also been associated with a more aggressive phenotype and reduced survival [40]. Additionally, INHBA inhibition has been shown to decrease interleukin-13 receptor alpha 2 (IL13Rα2) expression, which is associated with a more invasive phenotype and poorer prognosis [44,45]. Collectively, previous evidence suggests that miR-342-3p may exert its antitumor effects by interfering with the INHBA/IL13Rα2 axis and modulating TGF-β–dependent pathways involved in cellular proliferation and migration [40]. Consistent with this evidence, our study showed that an increase in the miR-342-3p in the BCS group following RET is compatible with a potentially favorable molecular response.

Similarly, miR-375-3p showed a nominally significant increase after 12 weeks of RET in both the HEA and BCS group (Time effect, *p* = 0.042), however, this effect did not remain statistically significant after BH-FDR correction (FDR-adjusted *p* = 0.073). Therefore, this finding should be interpreted cautiously. Nonetheless, this observation is relevant because several studies have reported downregulation of miR-375 in breast cancer, a condition associated with increased cellular proliferation, tamoxifen resistance, and epithelial–mesenchymal transition (EMT) [46,47,48,49]. In our study, baseline miR-375-3p expression was significantly higher in the HEA group than in BCS (*p* = 0.037) (Figure 1D). This difference may reflect underlying biological and therapeutic characteristics of the two groups, particularly considering the close relationship reported between miR-375 and estrogen receptor alpha (ERα) signaling. Previous evidence has described a positive feedback loop between miR-375 and ERα, supporting an interaction between miR-375 expression and estrogen signaling [47,50]. In this context, endocrine therapy may have modulated the ERα pathway and contributed to the baseline expression pattern observed in the BCS group, however, this present data cannot establish this mechanism.

The increase observed after 12 weeks of RET may reflect a beneficial adaptive response aimed at restoring tumor-suppressive function, however, it cannot be interpreted as restoration of tumor-suppressive function based on the present data alone. Indeed, this microRNA inhibits the expression of metadherin (MTDH) and Twist-related protein 1 (TWIST1), thereby reducing the invasive capacity and endocrine therapy resistance of breast cancer cells [51]. Given that all BCS included in this analysis exhibited an estrogen receptor–positive (ER^+^) tumor phenotype and were receiving endocrine therapy (tamoxifen, n = 3; aromatase inhibitors, n = 2), RET-induced re-expression of miR-375 acquires clinical relevance. Previous studies have demonstrated that reduced miR-375 levels are associated with tamoxifen resistance, mediated by activation of MTDH and EMT pathways [51,52]. Conversely, restoring its expression sensitizes cells to tamoxifen, promotes apoptosis, and reduces tumor proliferation, suggesting that RET may act as a complementary epigenetic modulator that enhances endocrine therapy efficacy [51]. Thus, our findings raise the hypothesis that RET-associated changes in circulating miR-375-3p may influence molecular pathways involved in endocrine therapy response, but whether these circulating changes influence treatment sensitivity requires direct functional investigation.

In contrast, no significant changes were detected in the expression of the selected oncomiRs miR-155-5p, miR-21-5p, and miR-221-3p following RET in either group (Figure 2). These findings differ from those reported by Alizadeh et al. (2019), who documented a significant reduction in these miRNAs after 12 weeks of high-intensity interval training (HIIT) combined with endocrine therapy [34]. This discrepancy likely depends on the physiological stimulus: HIIT activates metabolic stress and systemic inflammatory pathways (NF-κB, STAT3),whereas RET primarily stimulates mechanical anabolic signaling (mTOR, IGF-1), with a comparatively less inflammatory epigenetic impact [17]. Furthermore, participants in the study by Alizadeh et al. (2019) had recently completed antineoplastic treatment and displayed greater baseline molecular dysregulation, whereas our participants (mean survivorship of 4 years) showed more stable oncomiR levels, which could potentially contribute to the different responses observed between studies [34].

Regarding miRNAs associated with skeletal muscle homeostasis, miR-486-5p expression did not change significantly despite the observed increase in lower-limb skeletal muscle mass in both groups (Figure 3). This result does not necessarily indicate the absence of a molecular response, as miR-486-5p exerts a context-dependent dual role: it functions as an anabolic modulator of skeletal muscle through inhibition of PTEN and FOxO1, activating the IGF-1/PI3K/Akt/mTOR pathway, and simultaneously acts as a tumor suppressor, whose loss has been associated with increased proliferation and metastasis [53,54]. In this context, the post-RET stability of miR-486-5p may reflect a favorable adaptive equilibrium that preserves tumor-suppressive functions without compromising exercise-induced muscular adaptations. Similarly, miR-206 did not change significantly, although the BCS group showed a greater upward trend (+20%) that the HEA group (+2%). miR-206, an essential myomiR, regulates muscle differentiation and tissue regeneration through modulation of IGF-1 and activation of the PI3K/Akt/mTOR pathway [55]. Additionally, it functions as a tumor suppressor by targeting neurogenic locus notch homolog protein 3 (Notch3), inducing apoptosis and reducing the migration and invasion of breast cancer cells [56]. Given the lack of a statistically significant change, the observed numerical increase in BCS should be interpreted cautiously and considered hypothesis-generating rather than evidence of a protective adaptive modulation.

**Figure 4:**
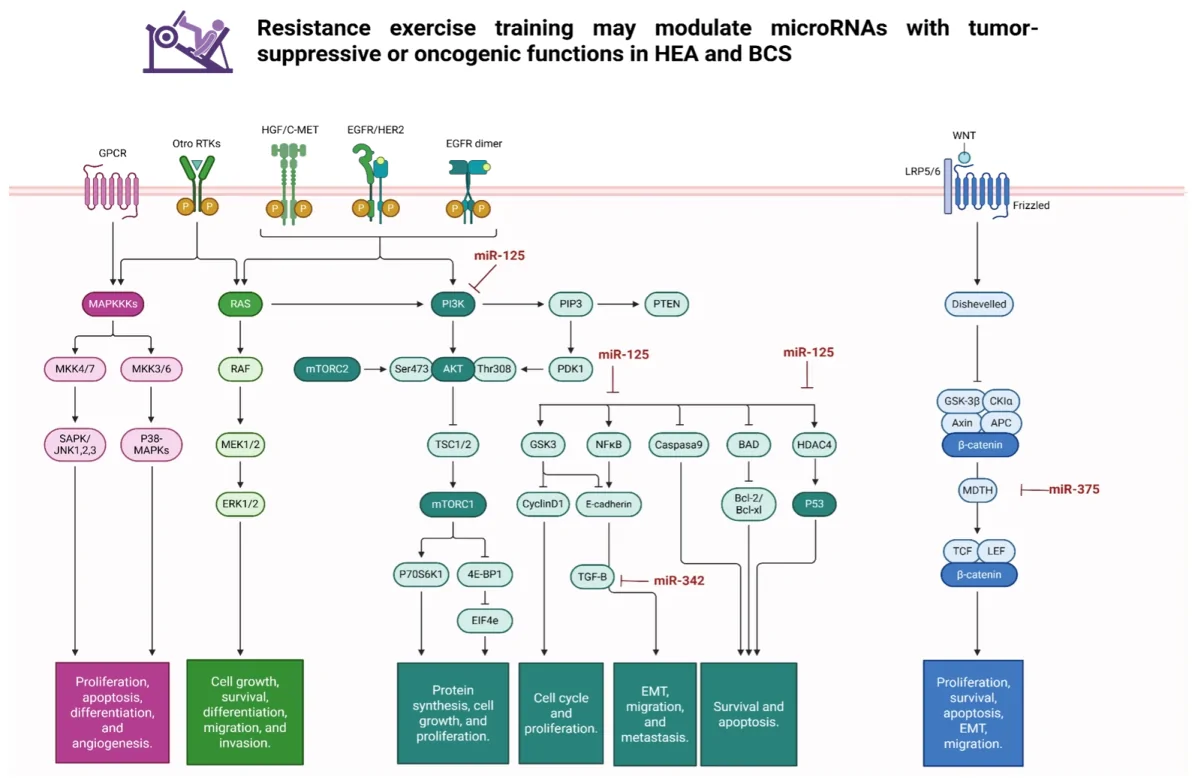
Twelve weeks of resistance exercise training may modulate microRNAs that act as tumor suppressors by participating in the inhibition of oncogenes in healthy postmenopausal women (HEA) and breast cancer survivors (BCS). GPCR: G protein–coupled receptor; MAPKK5: mitogen-activated protein kinase kinase 5; MKK4/7: MAP kinase kinase 4/7; SAPK/JNK1/2/3: stress-activated protein kinases/c-Jun N-terminal kinases 1, 2, and 3; MKK3/6: MAP kinase kinase 3/6; MAPKs: mitogen-activated protein kinases; RTKs: receptor tyrosine kinases; RAS: small G protein family (KRAS, NRAS, HRAS); RAF: RAF serine/threonine kinases; MEK1/2: mitogen-activated protein kinase kinases (MAP2K); ERK1/2: serine/threonine kinases; HGF: hepatocyte growth factor; c-MET: c-MET receptor tyrosine kinase; EGFR: epidermal growth factor receptor (HER1/ERBB1); HER2: human epidermal growth factor receptor 2; PI3K: phosphatidylinositol 3-kinase; PIP3: phosphatidylinositol-3,4,5-trisphosphate; PTEN: phosphatase and tensin homolog; PDK1: phosphoinositide-dependent kinase 1; Thr308: threonine 308; AKT: protein kinase B; Ser473: serine 473; mTORC2: mechanistic target of rapamycin complex 2; TSC1/2: tuberous sclerosis complex 1 and 2; mTORC1: mechanistic target of rapamycin complex 1; p70S6K1: ribosomal protein S6 kinase; 4E-BP1: eukaryotic translation initiation factor 4E–binding protein 1; eIF4E: eukaryotic translation initiation factor 4E; GSK3: glycogen synthase kinase 3; NF-κB: nuclear factor kappa B; TGF-β: transforming growth factor beta; BAD: BCL2-associated agonist of cell death; Bcl-2: anti-apoptotic Bcl-2 family protein; Bcl-xL: anti-apoptotic Bcl-2 family isoform; HDAC4: histone deacetylase 4; p53: tumor protein p53; WNT: Wnt protein family; LRP5/6: low-density lipoprotein receptor–related protein 5 and 6; GSK-3β: glycogen synthase kinase 3 beta; CK1α: casein kinase 1 alpha; MTDH: metadherin; TCF: T-cell factor; LEF: lymphoid enhancer-binding factor. Created with BioRender.com and adapted by the authors.

Our findings suggest that RET were associated with selective changes in the circulating expression of specific miRNAs with previously described tumor-suppressive functions. This molecular profile supports the therapeutic potential of resistance exercise as a complementary intervention in ER^+^ breast cancer survivors, alongside its established benefits for skeletal muscle preservation, and suggests potential associations with epigenetic regulation and endocrine therapy-related pathways. These findings provide promising preliminary evidence and a biological basis for future mechanistic studies investigating the potential role of exercise-associated circulating miRNAs in tumor-suppressive signaling (Figure 4).

Several limitations of the present study warrant considered. The findings of the present study cannot be extrapolated to premenopausal women or to those undergoing active primary oncologic treatment such as chemotherapy or radiotherapy due to substantial differences in the hormonal milieu, metabolic status, inflammatory burden, and treatment-induced molecular mechanisms, all of which may influence both circulating miRNA expression profiles and the biological response to RET. A further limitation is the heterogeneity of endocrine therapy within the BSC group (tamoxifen, n = 2; aromatase inhibitors, n = 3). Given that these therapies differentially affect estrogen signaling and exert distinct effects on bone mineral density and skeletal muscle homeostasis, they may also differentially regulate muscle-related circulating miRNAs, potentially contributing to the interindividual variability observed in the present study [9,57]. Although the sample size was limited, post hoc analysis demonstrated 95% statistical power, supporting the robustness of clinical outcomes related to skeletal muscle mass [21]. Nevertheless, the small sample size and participant selection for the present exploratory analysis using group-specific criteria should be considered when interpreting the molecular findings. Specifically, we selected BCS participants prioritizing clinical homogeneity and ongoing endocrine therapy, whereas we selected HEA participants based on responsiveness to exercise. These group-specific selection criteria may limit direct between-group comparisons; therefore, the present findings should be interpreted as exploratory and hypothesis-generating. Future studies should include more participants to analyze differences by type and duration of endocrine therapy among survivors.

Furthermore, using total plasma for miRNA analysis, although clinically relevant, does not allow identification of the specific tissue origin of circulating molecules. Because we did not assess extracellular vesicles, tissue-specific miRNA expression, target-gene regulation, or downstream functional effects, we cannot attribute the observed circulating miRNA changes to a specific tissue or interpreted them as direct evidence of an antitumor mechanism. Future investigations should incorporate isolation of muscle-derived exosome microRNAs and high-throughput sequencing to expand the repertoire of exercise-modulated molecules and further elucidate mechanisms of intercellular communication and muscular adaptation in breast cancer survivors.

In conclusion, this study showed that RET selectively modulated circulating miRNAs associated with tumor and muscle regulation. In BCS undergoing endocrine therapy, miR-125a-5p and miR-342-3p (tumor suppressors) showed increased responses to RET, with significant group × time interactions that remained significant after BH-FDR correction, along with upward trends in miR-206-3p and miR-486-5p (muscle regulators). Moreover, both groups exhibited a nominal increased in miR-375-3p expression, also considered a tumor suppressor, although this effect did not remain significant after FDR correction. These preliminary findings support the hypothesis that resistance exercise functions as an epigenetic modulator with clinical potential to preserve skeletal muscle mass while contributing to tumor suppression.

## 4. Materials and Methods

### 4.1. Experimental Design

**Figure 5:**
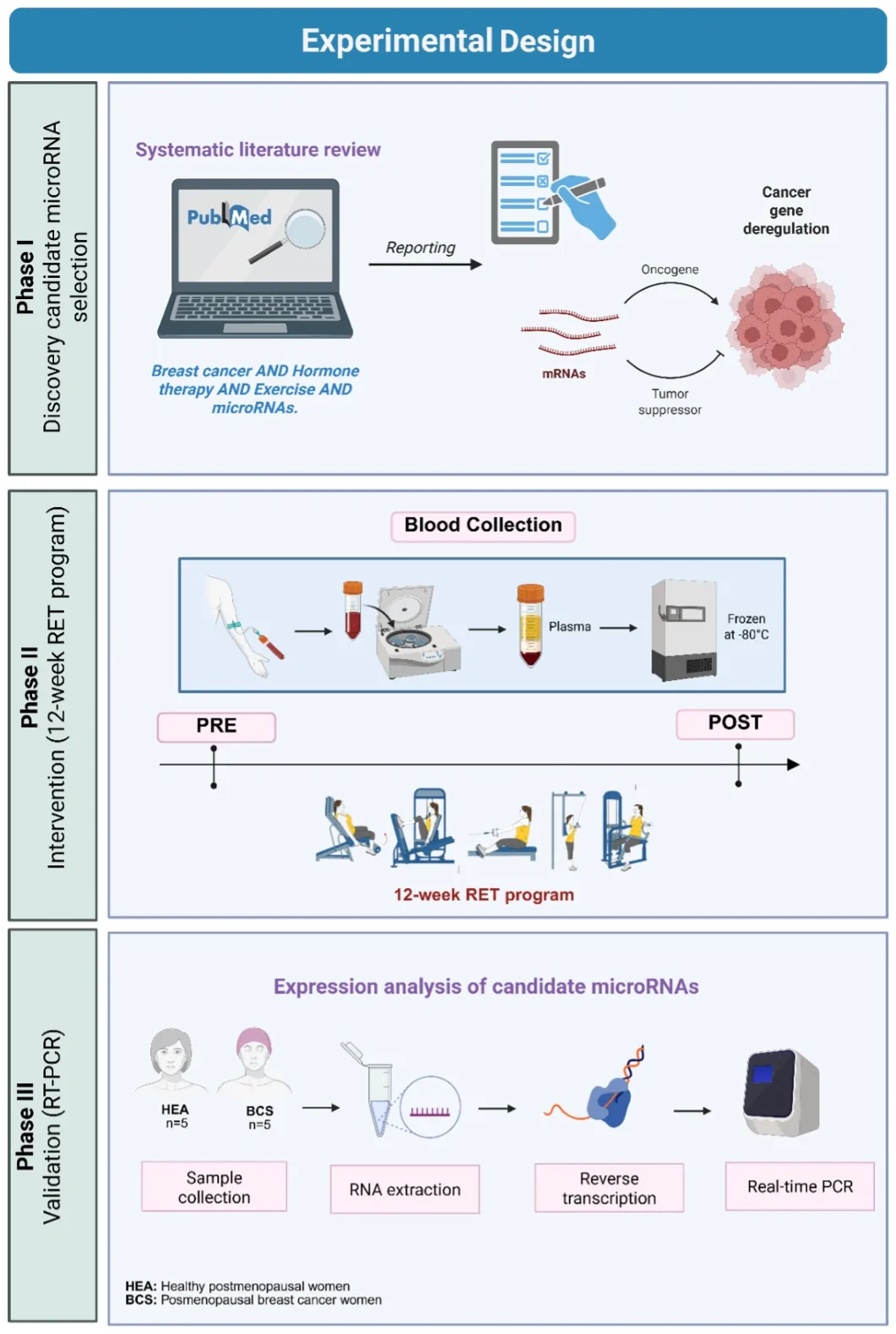
Analysis of miRNAs involved in the modulation of tumor and muscle homeostasis. Created with BioRender.com.

Our study was conducted in three phases (Figure 5):

1. *Discovery phase:* We conducted atargeted literature review informed by a structured search strategy was conducted to identify circulating microRNAs (miRNAs) with reported tumor suppressor or oncogenic (oncomiR) roles in breast cancer. The search was performed in PubMed using the following query: (((microRNA OR miRNA) AND (breast cancer OR breast neoplasms)) AND (“tumor suppressor miRNAs” OR “oncomiR”)), with results sorted by most recent publications. This search yielded approximately 170 records, and we selected 10 studies based on predefined eligibility criteria. Eligible studies were required to be conducted specifically in the context of breast cancer, including studies involving patients, human biological samples, and/or experimental breast cancer cell models, and to provide evidence regarding the biological function and/or clinical relevance of the candidate miRNAs. Specifically, we considered studies that provided evidence of tumor-suppressive or oncogenic functions, cell proliferation, apoptosis, migration/invasion, tumor progression, prognosis, and/or response to or resistance to endocrine therapy (Supplementary Table S2). In parallel, we selected miRNAs involved in skeletal muscle homeostasis were selected based on a previously published review by our research group, focusing on miRNAs associated with skeletal muscle mass regulation, anabolic and catabolic signaling pathways, and exercise responsiveness [26]. This combined approach allowed us to prioritize candidate miRNAs associated with tumor suppressor genes, oncogenes, and skeletal muscle regulation, and to formulated specific hypotheses about their expected response to RET before qPCR analyses were performed and the experimental results were known (Table 2).
2. *Intervention phase:* This study represents a secondary exploratory analysis derived from a primary study that included 13 healthy postmenopausal women (HEAs) and 11 breast cancer survivors (BCSs), in which the effects of a 12-week whole-body progressive RET program on skeletal muscle mass, muscle strength and functionality were evaluated and have been previously published [21,26,58]. Given the exploratory nature of the present molecular analysis and the limited availability of stored biological samples and analytical resources, we assessed miRNA expression in a subset of five participant from each group.

Participant selection followed group-specific criteria. In the HEA group, we included participants with the greatest exercise response, as defined by improvements in lower-limb strength and quadriceps muscle thickness, were included. In the BCS group, we prioritized clinical homogeneity and selected five postmenopausal participants undergoing adjuvant endocrine therapy (tamoxifen or aromatase inhibitors) for at least six months to minimize clinical heterogeneity related to oncological treatment.

Given these group-specific selection criteria, between-group comparisons should be interpreted within the exploratory and hypothesis-generating nature of the present molecular analysis. Training responses of the participants included in the miRNA analysis are provided in Supplementary Tables S3–S5 training responses for the participants included in the miRNA analysis, while the corresponding outcomes for the original cohort have been previously reported [21,58,59].

Participants completed a supervised, whole-body progressive RET program lasting 12 weeks, comprising 36 training sessions performed three times per week on non-consecutive days. Each session began with a 5-min warm-up on a cycle ergometer and active mobility exercises, followed by lower- and upper-body strength exercises performed on specific resistance machines. Lower-body training included one warm-up set followed by four training sets of leg press and leg extension exercises. Upper-body training consists of three sets each of chest press, elbow extension and horizontal row. A 2-min passive recovery interval was provided between sets. The session concluded with general stretching exercises. Training loads were prescribed at 60–80% of the one-repetition maximum (1RM), which was reassessed at week 6 to adjust training intensity. Adherence was defined as attendance at ≥80% of the scheduled training sessions (≥29 of 36 sessions).

3. *Sample preparation and determination of differential plasma miRNA expression:* Blood samples were collected by venipuncture from the antecubital vein in the morning after a 10–12 h overnight fast. We collected samples 48 h before the first training session and 48 h after the last session of the RET program. The 48-h post-intervention interval was selected to minimize the influence of acute and transient exercise-induced responses and to assess circulating miRNAs under resting post-training conditions [60]. Blood was drawn into EDTA-coated tubes and centrifuged at 2500 rpm for 15 min. The resulting plasma was aliquoted into microtubes and stored at −80 °C until analysis [61].

In the expression analysis phase, we assessed changes in candidate miRNA expression were assessed before and after the intervention using quantitative reverse transcription polymerase chain reaction (qRT-PCR). Pre- and post-intervention miRNA expression profiles were compared within and between groups to identify miRNAs differentially expressed in response to RET. In parallel, we explored the biological pathways regulated by these miRNAs were explored to gain insight into potential molecular mechanisms by which exercise may modulate tumor-related and muscle-regulatory processes, drawing on previously published evidence from the scientific literature.

Total RNA, including small RNAs, was isolated from 200 μL of plasma using the miRNeasy Serum/Plasma Kit (Qiagen, Hilden, Germany), according to the manufacturer’s instructions. Plasma samples previously stored at −80 °C were thawed on ice and centrifuged at 4000 rpm for 5 min before RNA extraction to remove residual cellular debris. The concentration of the extracted RNA was determined by fluorometry using a Quantus™ Fluorometer (Promega corporation, Madison, WI, USA) and the QuantiFluor^®^ RNA System according to the manufacturer’s instructions. Subsequently, 1pg of total RNA was reverse transcribed into complementary DNA (cDNA) using the miRCURY LNA RT Kit (Qiagen, Hilden, Germany), following the manufacturer’s protocol.

We quantified miRNAs using a two-step qRT-PCR approach with the miRCURY LNA SYBR^®^ Green PCR Kit (Qiagen, Hilden, Germany), according to the manufacturer’s instructions. Supplementary Table S6 provides the corresponding GeneGlobe IDs and catolog numbers for each mIRNA. Primer sequences are proprietary and therefore not publicly available. Amplification conditions included an initial activation step at 95 °C, followed by 40 cycles of denaturation at 95 °C for 10 s and combined annealing/extension at 56 °C for 60 s, ending with a melting curve analysis. All reactions were performed in duplicate.

We initially evaluated three candidate references RNAs: SNORD48, hsa-miR-16-5p, and hsa-miR-24-3p as reference genes.

SNORD48 did not amplify reliably under the experimental conditions and was therefore excluded from subsequent stability assessment. The expression stability of t hsa-miR-16-5p, and hsa-miR-24-3p, was evaluated using RefFinder online tool. The comprehensive reference-gene stability assessment tool that integrates geNorm, NormFinder, Bestkeeper version 1, and comparative CT method to provide an overall stability ranking of candidate reference genes [62,63]. Based on the stability across the experimental samples and conditions, hsa-miR-16-5p showed greater expression stability than hsa-miR-24-3p and was selected as the endogenous control for normalization. The stability assessment of the candidate endogenous control is provided in Supplementary Table S6. Relative miRNA expression levels were calculated using the 2^−ΔΔCt^ method [62,63].

**Table 2:** MicroRNAs involved in muscle and/or tumor regulation with potential modulation by resistance exercise training.

miRNA (miR)	Function	Plasma Expression Levels	Hypothesis
miR 206-3p [56]	miR-206-3p is a skeletal muscle–specific microRNA (myomiR) that plays a key role in myogenesis and myoblast differentiation. It negatively regulates genes that inhibit differentiation, such as HDAC4, and components of the Notch signalling pathway (NOTCH3). It has also been reported to silence IGF-1 and Pax7, thereby modulating the balance between proliferation and differentiation. In the tumor context, it acts as a tumor suppressor by repressing genes associated with cell proliferation and migration (e.g., MET, KRAS).	Plasma miR-206 levels are typically elevated under conditions of muscle atrophy, such as cancer, aging, or disuse, reflecting catabolic processes associated with loss of muscle mass and strength. In contrast, its expression is reduced in several malignant neoplasms, which is associated with tumor progression and poorer clinical prognosis.	Increases after 12 weeks of resistance exercise training (tumor suppressor) ^+^.
miR 486-5p [64]	miR-486 is associated with the regulation of muscle growth and the differentiation of muscle cells. It negatively regulates PTEN and FOXO1, thereby activating the PI3K/Akt pathway in cardiac and skeletal muscle.	Increased miR-486 levels are associated with enhanced muscle regeneration and attenuation of atrophy, contributing to the maintenance or improvement of muscle mass and physical function. Conversely, reduced levels of this miRNA have been described in conditions of muscle atrophy, favoring the loss of muscle mass and strength.	Increases after 12 weeks of resistance exercise training ^+^.
**microRNAs involved in the regulation of tumor homeostasis (tumor suppressors)**			
**miRNA (miR)**	**Function**	**Plasma expression levels**	**Hypothesis**
miR Let-7f-5p [65]	Let-7f primarily acts as a tumor suppressor, as it negatively regulates the expression of multiple oncogenic genes. Its targets include RAS, MYC, HMGA2, STAT3, and VEGF, which are involved in cell proliferation, tumor survival, angiogenesis, and epithelial–mesenchymal transition (EMT). Consequently, reduced Let-7f expression in breast cancer is associated with increased tumor aggressiveness, metastatic progression, and poorer clinical prognosis.	Low Let-7f expression in breast cancer has been associated with worse prognosis and greater tumor aggressiveness. Therefore, Let-7f is not only crucial for regulating tumor behavior but may also serve as a potential biomarker for breast cancer progression and treatment response.	Increases after 12 weeks of resistance exercise training ^+^.
miR 125 a-5p [36]	miR-125a-5p is a microRNA that primarily functions as a tumor suppressor across multiple cancer types. One of its major targets is histone deacetylase 4 (HDAC4), whose inhibition leads to reduced tumor growth and cellular motility. By suppressing HDAC4, miR-125a-5p promotes histone acetylation, which increases the expression of pro-apoptotic genes such as BAX (BCL2-associated X protein) and BID (BH3-interacting domain death agonist), while decreasing the expression of anti-apoptotic genes such as BCL2 (B-cell lymphoma 2), thereby favoring apoptosis and limiting cancer cell survival.	miR-125a-5p has been identified as a prognostic biomarker that suppresses breast cancer tumorigenesis. Reduced circulating expression is associated with lower survival and correlates negatively with tumor size, lymph node status, and tumor grade.	Increases after 12 weeks of resistance exercise training ^+^.
miR 342-3p [40,66]	miR-342 is prominently expressed in luminal breast tumors and is positively associated with estrogen receptor (ER)-positive status. It has been shown to be a significant biomarker for distinguishing breast cancer subtypes and for supporting disease diagnosis. miR-342 acts as a tumor suppressor, exerting antitumor effects by negatively regulating genes and pathways involved in cell proliferation, survival, and metastasis. Specifically, miR-342 inhibits PIK3CA (phosphatidylinositol-4,5-bisphosphate 3-kinase catalytic subunit alpha) within the PI3K/Akt pathway, thereby reducing cell proliferation and survival. Additionally, it negatively regulates pro-metastatic genes such as MMP9 (matrix metalloproteinase 9), VEGF, ZEB1, CXCR4 (C–X–C motif chemokine receptor 4), and SNAI1 (Snail family transcriptional repressor 1), thus limiting tumor invasion and metastasis.	Its expression correlates positively with ER-positive status and with improved sensitivity to hormonal therapies, making it an important diagnostic biomarker in breast cancer with estrogen receptor positivity.	Increases after 12 weeks of resistance exercise training ^+^.
miR 375-3p [51]	miR-375 acts as a tumor suppressor, regulating cell proliferation and promoting apoptosis, primarily through modulation of the p53 pathway. This miRNA inhibits the expression of genes that suppress p53 activity, such as MDM2(Mouse Double Minute 2 homolog), thereby enabling p53 activation and apoptosis in tumor cells. In addition, miR-375 negatively regulates proliferative genes such as JAK2(Janus kinase 2), a key component of the JAK/STAT pathway, leading to reduced cell proliferation. miR-375 has also been associated with improved response to tamoxifen in patients with estrogen receptor–positive (ER+) breast cancer, suggesting a role in modulating sensitivity to hormonal therapy.	In ER+ breast cancer, miR-375 is generally overexpressed. Elevated circulating levels have been correlated with the presence of this subtype, supporting its potential utility in early detection and patient stratification.	Increases after 12 weeks of resistance exercise training ^+^.
**microRNAs involved in the regulation of tumor homeostasis (oncomiRs)**			
**miRNA (miR)**	**Function**	**Plasma expression levels**	**Hypothesis**
miR 155-5p [67,68]	miR-155 is a microRNA that plays a crucial role in determining breast cancer prognosis by acting as an oncogene. When overexpressed, miR-155 negatively regulates several tumor suppressor genes, such as PTEN and SOCS1 (Suppressor of Cytokine Signaling 1), as well as other negative regulators of the cell cycle and apoptosis, including TGF-β (Transforming Growth Factor-beta) and SMAD5. This inhibition promotes cellular proliferation and tumor survival, thereby contributing to cancer development and progression.	Overexpression of miR-155 is associated with an unfavorable prognosis, including increased tumor aggressiveness and reduced survival. It has been linked to the downregulation of tumor suppressor genes and the promotion of invasion and metastasis. High miR-155 levels correlate with a greater likelihood of recurrence and poorer survival in patients with breast cancer.	It decreases following 12 weeks of resistance training ^+^.
miR 21-5p [69]	It is one of the most extensively studied microRNAs in breast cancer due to its relevance to prognosis. It functions as an oncogene by inhibiting tumor suppressor genes such as PTEN (Phosphatase and Tensin Homolog) and PDCD4 (Programmed Cell Death 4).	Elevated levels of miR-21 are associated with poor prognosis, including higher recurrence rates and reduced overall survival. miR-21 expression correlates with chemoresistance and tumor progression.	It decreases following 12 weeks of resistance training ^+^.
miR 221-3p [70,71]	This microRNA influences breast cancer prognosis by promoting cellular proliferation and resistance to apoptosis in cancer cells. It functions as an oncogene by inhibiting tumor suppressor genes such as PTEN and CDKN1B (Cyclin-Dependent Kinase Inhibitor 1B). In addition, it increases the expression of VEGF (Vascular Endothelial Growth Factor) and activates NF-κB (Nuclear Factor kappa-light-chain-enhancer of activated B cells), thereby contributing to tumor angiogenesis and the expression of anti-apoptotic genes.	Overexpression of miR-221 is associated with a poorer prognosis, including resistance to hormonal therapy and reduced survival. Its overexpression correlates with worse clinical outcomes and an increased risk of recurrence, particularly in patients receiving tamoxifen treatment.	It decreases after 12 weeks of resistance training ^+^.

^+^ Hypotheses for each candidate miRNA were established a priori based on the literature review, before qPCR analysis and knowledge of the experimental results.

### 4.2. Statistical Analysis

All data were processed using SPSS version 25.0 (IBM Corp., Armonk, NY, USA) and Prism version 8 (GraphPad, CA, USA). We assessed variable normality using the Shapiro-Wilk test, and all variables were normally distributed. Continuous variables are therefore presented as mean ± standard deviation (SD). Two-way repeated measures analysis of variance (ANOVA) was performed to assess main effects and interaction effects of group and time and the group x time interaction. When a significant group × time interaction was detected, we conducted post hoc comparisons using Student’s *t*-tests: paired *t*-tests for within-group comparisons (PRE vs. POST) and independent *t*-tests for between-group comparisons. Additionally, percentage change (Δ%) was calculated for each variable based on pre- and post-intervention values, and between-group differences in these relative changes were analyzed using independent Student’s *t*-tests. For miRNAs with statistically significant effects, we apllied Benjamini–Hochberg false discovery rate (BH-FDR) correction was applied within their predefined functional category to account for multiple testing. All statistical analyses were two-tailed, and significance was set at *p* < 0.05.

## Figures and Tables

**Table 1:** Participant’s physical characteristics.

	HEA (n = 5)	BCS (n = 5)	*p*-Value
Age (years)	55 ± 3	53 ± 1	0.213
Body mass (kg)	61.4 ± 9.5	68.4 ± 5.9	0.201
Height (cm)	153 ± 6.1	156 ± 4.5	0.254
BMI (kg·m^−2^)	25.2 ± 1.8	25.2 ± 1.3	0.940
HR (beats·min^−1^)	74 ± 7.8	72 ± 11.3	0.801
SBP (mm Hg)	113.4 ± 19.3	115.1 ± 10.1	0.840
DBP (mm Hg)	77.2 ± 11.9	78.2 ± 8.2	0.441

Data are presented as mean ± SD. HEA: healthy postmenopausal women group; BCS: breast cancer survivor group; BMI: body mass index; SBP: systolic blood pressure; DBP: diastolic blood pressure; HR: heart rate; beats·min^−1^: beats per minute. Data were analyzed using an independent-samples *t*-test.

## Data Availability

The datasets generated and analyzed during the current study are not publicly available because the informed consent signed by the participants did not include permission for unrestricted public data sharing. The data associated with the paper are not publicly available but are available from the corresponding author on reasonable request and subject to approval by Ethics Committee.

## Supplementary Materials

The following supporting information can be downloaded at: https://www.mdpi.com/article/10.3390/ijms27177900/s1.
