# Supplementary material for: Resistance Exercise Training Selectively Modulates Tumor Suppressor and Muscle-Regulatory MicroRNAs in Breast Cancer Survivors and Healthy Postmenopausal Women: An Exploratory Study
Source: Int J Mol Sci. 2026 Sep 4;27(17):7900. doi: 10.3390/ijms27177900 (PMC13566732; doi:10.3390/ijms27177900)
Supplement: Supplementary file 1 [file ijms-27-07900-s001.zip › ijms-4481744-supplementary.pdf]

Supplementary Table S1. Nominal and Benjamini–Hochberg FDR-adjusted P-values for miRNA analyses

**miRNA**	**Functional category**	**Time effect *P***	**Time FDR-adjusted *P***	**Group x Time effect *P***	**Group x Time effect FDR-adjusted *P***
let-7f-5p	Tumor suppressor	0.087	0.087	0.534	0.534
miR-125a-5p	Tumor suppressor	0.055	0.073	**0.017**	**0.042**
miR-342-3p	Tumor suppressor	**0.032**	0.073	**0.021**	**0.042**
miR-375-3p	Tumor suppressor	**0.042**	0.073	0.412	0.534

Nominal *P*-values represent the unadjusted results obtained from the repeated-measures ANOVA (time and group × time effects). FDR-adjusted *P*-values were obtained using the Benjamini–Hochberg false discovery rate (BH-FDR) correction within the predefined tumor-suppressor miRNA category. Bold values indicate statistical significance (***P* < 0.05**). FDR: false discovery rate.

Supplementary Table S2. Studies supporting candidate miRNA selection during the discovery phase

miRNA	Selected study/ studies	Study model	Key finding relevant to candidate selection	Rational for inclusion
miR-206-3p	Song et al., 2009 [57]	Breast cancer cells	miR-206 targeted NOTCH3, promoted apoptosis, and inhibited tumor cell migration and focus formation, supporting its tumor-suppressive activity.	Selected because of its dual relevance as a skeletal muscle-related miRNA (myomiR) and its reported tumor-suppressive role in breast cancer.
miR-486-5p	Gharehdaghchi et al., 2020 [65]	Breast cancer cells.	miR-486-5p was associated with the regulation of breast cancer cell proliferation and migration.	Selected because of its established role in skeletal muscle growth/homeostasis and its additional relevance to breast cancer cell behaviour.
let-7f-5p	Qattan et al., 2017 [66]	Plasma from women with breast cancer.	Members of the let-7 family showed robust circulating expression in breast cancer patients, supporting their relevance as circulating tumor-suppressor miRNAs.	Selected as a circulating tumor-suppressor miRNA with direct evidence in breast cancer patients.
miR-125a-5p	Hsieh et al., 2015 [36]	Breast cancer patients and experimental breast cancer models.	miR-125a-5p acted as a tumor suppressor through HDAC4 regulation. Reduced expression was associated with adverse clinicopathological characteristics and poorer prognosis.	Selected because of its tumor-suppressive and prognostic relevance in breast cancer, including evidence supporting its potential as a circulating biomarker.
miR-342-3p	Young et al., 2017 [67]; Liu Q et al., 2022 [40]	Breast cancer patients / ER-positive breast cancer.	miR-342 expression was associated with ER-positive breast cancer, tamoxifen sensitivity, and breast cancer survival.	Selected because of its tumor-suppressive role and particular relevance to ER-positive breast cancer and endocrine therapy response.
miR-375-3p	Ward et al., 2013 [52]	ER-positive breast cancer cells / tamoxifen-resistant models.	Re-expression of miR-375 reversed tamoxifen resistance and associated EMT-like properties in breast cancer cells.	Selected because of its relationship with breast cancer biology and, particularly, endocrine therapy sensitivity/resistance in ER-positive disease.

miR-155-5p	Xu et al., 2025 [68]; Abdel-Samed et al., 2024 [69]	Breast cancer cells/exosomal models and breast cancer patients.	miR-155-5p was associated with breast cancer proliferation and metastasis through NF-κB-related mechanisms. Circulating miR-155 was also investigated as a biomarker in breast cancer patients.	Selected as an oncomiR because of its association with tumor-promoting pathways, proliferation, metastasis, and its potential relevance as a circulating breast cancer biomarker.
miR-21-5p	Yan et al., 2008 [70]	Human breast cancer tissue/patients.	miR-21 overexpression was associated with advanced clinical stage, lymph node metastasis, and poorer prognosis in breast cancer.	Selected as a well-established breast cancer-related oncomiR associated with tumor progression and adverse clinical outcomes.
miR-221-3p	Patellongi et al., 2023 [71]; Amiruddin et al., 2022 [72]	Patients with luminal breast cancer receiving tamoxifen.	Circulating miR-221/222 expression was investigated as a predictor of tamoxifen treatment outcome, and miR-221 was associated with tamoxifen resistance in luminal breast cancer.	Selected as an oncomiR with relevance to endocrine therapy response and tamoxifen resistance in luminal breast cancer.

miRNA: microRNA, oncomiR: oncogenic microRNA, myomiR: muscle-specific or muscle-enriched microRNA, Erα: estrogen receptor Alpha, ER+: estrogen receptor-positive, EMT: epithelial–mesenchymal transition, RET: resistance exercise training, BCS: breast cancer survivors group, HEA: healthy postmenopausal group, HDAC4: histone deacetylase 4, NOTCH3: Notch receptor 3, NF-κB: nuclear factor kappa B.

Supplementary Table S3. Quadriceps muscle thickness before and after 12 weeks of resistance exercise training.

	HEA (*n*=5)		BCS (*n*=5)		STATISTICS (*P*-value)		
	PRE	POST	PRE	POST	Time	Time × Group	Group
Vastus intermedius muscle thickness (cm)	1.33±0.3	1.41±0.3	1.36±0.4	1.49±0.5	0.200	0.783	0.785
Rectus femoris muscle thickness (cm)	1.98±0.2	2.05±0.2	1.82±0.1	1.95±0.2	**0.003**	0.190	0.308
Total quadriceps muscle thickness (cm)	3.45±0.3	3.61±0.5	3.35±0.5	3.61±0.6	**0.032**	0.559	0.859

Data presented as mean±SD. HEA: Healthy group; BCS: breast cancer survivors group. Data were analyzed using repeated measures ANOVA (time x group). *Bold numbers at the $P < 0.05$ level.

Supplementary Table S4. Strength parameters before and after 12 weeks of resistance exercise training.

	HEA (*n*=5)		BCS (*n*=5)		STATISTICS (*P*-value)		
	PRE	POST	PRE	POST	Time	Time × Group	Group
1RM chest press (kg)	53.0±2.7	64.0±8.2	46.0±15.6	56.0±15.2	**<0.001**	0.771	0.329
1RM horizontal row (kg)	38.0±6.5	49.0±8.2	36.0±6.5	49.0±8.2	**<0.001**	0.724	0.597
1RM elbow extension (Kg)	27.0±4.4	33.0±5.7	25.0±5.0	34.0±6.5	**<0.001**	0.195	0.883
1RM leg extension (kg)	59.7±9.8	84.4±10.4	54.4±15.9	73.4±20.1	**<0.001**	0.255	0.388
1RM leg press (kg)	97.0±23.45	151.5±31.6*+	60.8±19.7	91.5±20.8*+	**<0.001**	**0.008**	**0.013**
Dominant Grip strength (kg)	27.8±4.4	29.1±5.9	26.6±7.5	29.6±6.8	**0.008**	0.213	0.933

Data presented as mean±SD. HEA: Healthy group; BCS: breast cancer survivors group; 1RM: 1-repetition maximum. Data were analyzed using repeated measures ANOVA (time x group). Post hoc comparisons were performed using independent-samples and paired-samples t-tests. *P = 0.029 for HEA vs. BCS at baseline and **P = 0.008 for HEA vs. BCS post-intervention (independent-samples t-test); †P < 0.001 for pre- vs. post-intervention in HEA and ‡P = 0.003 for pre- vs. post-intervention in BCS (paired-samples t-test).

Supplementary Table S5. Functional capacity and Physical performance before and after 12 weeks of resistance exercise training

	HEA (n=5)		BCS (n=5)		STATISTICS (*P*-value)		
	PRE	POST	PRE	POST	Time	Time × Group	Group
6MWT (m)	607.2±28	668.0±35	592.7±44	600.6±9	**0.020**	0.055	0.022
TUG (s)	6.5±0.7	5.3±0.6	6.2±0.7	5.8±0.6	**0.004**	0.067	0.807
4 m Walk time (s)	3.0±0.4	2.8±0.5	2.8±0.5	2.5±0.4	0.324	0.811	0.140
Walk speed (m/s)	1.3±0.2	1.4±0.3	1.5±0.3	1.6±0.3	0.361	0.828	0.109
Chair stand test (s)	9.2±0.7	7.8±1.7	9.5±1.5	8.0±1.6	**0.020**	0.846	0.712

Data presented as mean±SD. 6MWT: 6-minute walk test; HEA: Healthy group; BCS: breast cancer survivors group; TUG: Timed up and Go. Data were analyzed using repeated measures ANOVA (time x group). *Bold numbers at the $P < 0.05$ level.

Supplementary Table S6. miRNA assays used for RT-qPCR analysis, including GeneGlobe IDs and

catalog numbers

miRNA	GeneGlobe ID	Cat. No.
hsa-miR 206-3p	YP00206073	339306
has-miR 486-5p	YP00204001	339306
has-miR Let-7f-5p	YP00204359	339306
has-miR 125 a-5p	YP00204339	339306
has-miR 342-3p	YP00205625	339306
has-miR 375-3p	YP00204362	339306
has-miR 155-5p	YP02119311	339306
has-miR 21-5p	YP00204230	339306
hsa-miR 221-3p	YP00204532	339306
SNORD48	YP00203903	339306
hsa-miR 16-5p	YP00205702	339306
hsa-miR 24-3p	YP00204260	339306

Supplementary Table S7. Stability assessment of candidate endogenous controls for plasma miRNA normalization

**Candidate endogenous control**	**Amplification**	BestKeeper			geNorm	NormFinder	**Outcome**
		**Mean Ct**	**SD (Ct)**	**CV (%)**	**M value**	**Expression stability across experimental conditions.** Range of mean Ct (HEA/BCS; PRE/POST)	
hsa-miR-16-5p	Confiable	21.36	0.87	4.07	1.016	0.747	Selected
hsa-miR-24-3p	Confiable	24.60	1.04	4.23	1.016	1.639	Not selected
SNORD48	No confiable	--	--	--	--	--	Excluded

Ct values represent the mean of technical replicates. SNORD48 was excluded due to unreliable amplification. Lower SD and CV indicate greater expression stability. Because only two candidate miRNAs showed reliable amplification, geNorm yielded the same M value for both candidates and could not independently rank them. hsa-miR-16-5p showed lower Ct variability and greater consistency across experimental conditions and was therefore selected for normalization. HEA: healthy postmenopausal women group; BCS: breast cancer survivors group; PRE: pre-intervention; POST: post-intervention; Ct: cycle threshold; SD: standard deviation; CV: coefficient of variation.

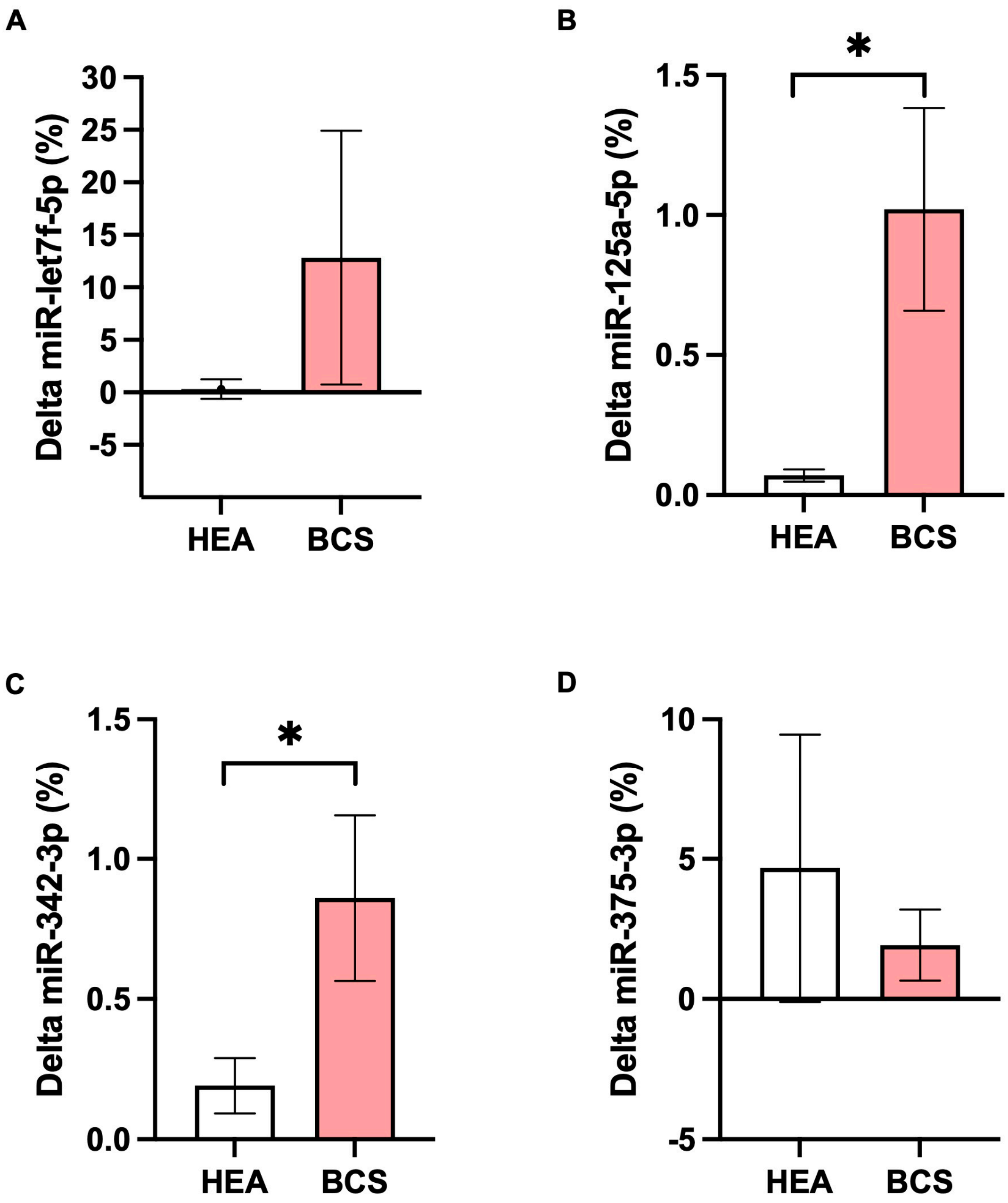

Figure supplementary S1. Percentage change (Δ%) between PRE and POST values was calculated for each variable: (A) miR-let-7f-5p, (B) miR-125a-5p, (C) miR-342-3p, and (D) miR-375-3p. Between-group differences in percentage changes were analyzed using independent Student's tests. Significant between-group differences were observed for miR-125a-5p (B) and miR-342-3p (C), as indicated by * ($P < 0.05$). No significant between-group differences were observed for miR-let-7f-5p (A) or miR-375-3p (D). Data are presented as mean ± SD.
